# Non-Detection of Human Herpesvirus 8 (HHV-8) DNA in HHV-8-Seropositive Blood Donors from Three Brazilian Regions

**DOI:** 10.1371/journal.pone.0023546

**Published:** 2011-08-08

**Authors:** José Eduardo Levi, Maria Claudia Nascimento, Laura Masami Sumita, Vanda Akico Ueda Fick de Souza, Wilton S. Freire, Philippe Mayaud, Claudio S. Pannuti

**Affiliations:** 1 Instituto de Medicina Tropical da Universidade de São Paulo, São Paulo, Brazil; 2 Departamento de Moléstias Infecciosas e Parasitárias (LIM 52 -HC), Faculdade de Medicina da Universidade de São Paulo, São Paulo, Brazil; 3 Faculty of Infectious and Tropical Diseases, London School of Hygiene and Tropical Medicine, London, United Kingdom; University of Cape Town, South Africa

## Abstract

Human herpesvirus 8 (HHV-8), also known as Kaposi's sarcoma-associated herpesvirus (KSHV), is the etiologic agent of all forms of Kaposi's sarcoma, primary effusion lymphoma and the plasmablastic cell variant of multicentric Castleman disease. In endemic areas of sub-Saharan Africa, blood transfusions have been associated with a substantial risk of HHV-8 transmission. By contrast, several studies among healthy blood donors from North America have failed to detect HHV-8 DNA in samples of seropositive individuals. In this study, using a real-time PCR assay, we investigated the presence of HHV-8 DNA in whole-blood samples of 803 HHV-8 blood donors from three Brazilian states (São Paulo, Amazon, Bahia) who tested positive for HHV-8 antibodies, in a previous multicenter study. HHV-8 DNA was not detected in any sample. Our findings do not support the introduction of routine HHV-8 screening among healthy blood donors in Brazil. (WC = 140).

## Introduction

Human herpesvirus 8 (HHV-8), also known as Kaposi's sarcoma-associated herpesvirus (KSHV), is the etiologic agent of all forms of Kaposi's sarcoma, primary effusion lymphoma and the plasmablastic cell variant of multicentric Castleman disease [1]. Detection of HHV-8 antibodies has been extensively used to determine the prevalence of the infection and to investigate routes of viral transmission. Initial studies have suffered from a wide variability of serological assays. Nevertheless, they uncovered the existence of different rates of HHV-8 worldwide. As expected, the highest prevalence rates were observed in areas where Kaposi's sarcoma (KS) was endemic, like in Eastern and Central Africa [2]. In these areas, seroprevalence in blood donors may be as high as 48%, as observed in Tanzania [3]. This contrasts with much lower rates found in US blood donors ranging from 2.8% to 7.3% [4]–[6]. Brazil may be considered a region of intermediate endemicity, as we have detected a HHV-8 seroprevalence of 25% among 3,493 blood donors from three different regions of the country [7].

Since HHV-8 has been causally linked to KS, concern was raised earlier on its potential transmission by blood transfusion and organ transplantation. Moreover, the detection of HHV-8 RNA in target cells inoculated with filtered fluids collected from activated CD19 cells of a healthy North American blood donor in 1997 fostered research on the possible transmission of this oncogenic virus by blood transfusion [8], analogous to the proven association between Human T-cell Lymphotropic Virus 1 (HTLV-1) and the development of leukemia among recipients of infected blood units.

Since then, a number of studies have addressed this important issue, with discordant results. In endemic areas of sub-Saharan Africa, blood transfusions have been associated with a substantial risk of HHV-8 transmission. For example, in Tanzania and Central African Republic, HHV-8 DNA was detected in 4.5% and 22.5% of blood donors, respectively [3]; [9]. In Uganda, HHV-8 seropositivity was shown to be significantly more frequent in transfused versus never-transfused children with sickle-cell disease [10], and recipients of HHV-8 seropositive blood units were at a significantly higher risk of seroconversion compared to recipients of seronegative blood units [11]. By contrast, several studies among healthy blood donors from North America using sensitive PCR assays have failed to detect HHV-8 DNA in samples of HHV-8 seropositive individuals [4]; [12]; [13]. A large cohort of donor-recipient pairs in the US did not identify any case of HHV-8 transmission [5], which was corroborated by findings of a similar study in Jamaica [14].

In one Brazilian study, HHV-8 antibodies were detected in 16/400 (4%) blood donors, one of whom was found to also harbor HHV-8 DNA in both peripheral blood mononuclear cells (PBMCs) and plasma [15]. So far, universal leukoreduction has not been implemented in the country, providing theoretical opportunities for transfusion-associated transmission of cell-associated viruses such as herpesviruses.

The aim of this study was to evaluate the prevalence of HHV-8 DNA in blood samples of apparently healthy HHV-8 seropositive blood donors to determine their potential for HHV-8 transmission.

## Materials and Methods

### Study sites

Brazil has a population of 190 million inhabitants, mainly composed of descendants of Caucasian, African and Amerindian indigenous populations, with a large degree of ethnic mixing. The majority of Caucasian descendants live in the Southern parts of the country, African descendants are ubiquitous with a large presence in the Northeast, whilst most indigenous Amerindian populations live in remote areas of the Central-Western and Northern regions. For this study, we included samples from the main governmental state blood banks located in the widely-separated cities of Manaus (Amazon state, North), Salvador (Bahia state, Northeast), and São Paulo (São Paulo state, Southeast).

### Study population, enrolment procedures and HHV-8 seropositive specimens

Frozen whole blood specimens were retrieved from a repository of blood samples obtained from voluntary first time blood donors who tested positive for HHV-8 antibodies using a whole cell ELISA. Those specimens were collected during a multicenter study conducted among blood donors from these three large urban centers of Brazil, which aimed to determine the seroprevalence, risk factors and molecular characterization of HHV-8 infection in these regions of Brazil [7]. The characteristics of the study population and enrolment procedures as well as a detailed description of the ELISA assays used for HHV-8 serological screening have been reported elsewhere [7], [16]. As reported, HHV-8 serum antibodies were detected by whole-virus ELISA in 878 of 3,493 (25%) screened blood donors.

### Ethical considerations

Eligible blood donors who consented to the study were asked to read an information sheet and sign a consent form giving permission for their samples to be tested for HHV-8, in addition to serological tests performed routinely in Brazilian blood banks, and to provide an extra 10 mL of EDTA treated blood sample for molecular studies on HHV-8.

The Institutional Review Boards of each blood bank (Research Ethics Committees of the Fundação de Hematologia e Hemoterapia da Bahia-HEMOBA, the Fundação de Hematologia e Hemoterapia do Amazonas (HEMOAm), and the Hospital das Clínicas da Faculdade de Medicina da Universidade de São Paulo (HEMOSP)), as well as the Ethics Board of the Brazilian Ministry of Health (CONEP) and the Research Ethics Committee of the London School of Hygiene & Tropical Medicine approved the study protocol.

### HHV-8 molecular assays

*DNA extraction*. DNA was extracted from frozen EDTA-treated whole blood (200 µL), using QIAmp DNA Blood Mini kit (Qiagen, Brazil) and eluted into 100 µL of the elution buffer provided in the kit. DNA concentration was measured in a subset of 300 randomly chosen samples (100 from each center) by spectrophotometry at 260 nM in the NanoDrop (Thermo Scientific NanoDrop Products-USA) system. Values ranging between 1 and 160 ng/µL (mean 40 ng/µL, SD ±23,6 ng/µL; median37 ng/µL), were verified, while three samples (1%) had no OD values. The eluate volume input in the real-time PCR was 5 µL for all samples, resulting in a human DNA input in between 5 and 800 ng, corresponding roughly to 75 to 121,212 diploid cells (mean 6,060 cells). DNA quality was evaluated by PCR amplification of the human β-globin gene, prior to investigation of HHV-8 DNA.

### ORF 26 real-time PCR

#### Sensitivity

Analytical sensitivity of this assay was obtained by using a plasmid containing an insert of 233 base pairs from the minor capsid ORF26 cloned into pGEMT-easy vector (Promega- 2800 Woods Hollow Road Madison-USA). The designed primers (Table 1) amplified a 66 bp fragment from the cloned amplicon. The DNA concentration of a pure preparation of the plasmid was determined by spectrophotometry to contain 68.47 µg/ml, corresponding to 1.95×10^13^ copies/mL. This plasmid solution was serially diluted on fresh human blood seronegative and PCR negative for HHV-8, in order to obtain a dilution series of HHV-8 DNA from 1.95×10^4^ to 10^0^ copies/mL of whole blood. Ten replicates were extracted from each dilution point and submitted to a real-time PCR reaction containing 400 ηM of forward and reverse primers and 160 ηM of the probe (Table 1), in addition to 12.5 µL of 2X TaqMan Universal PCR Master Mix (Applied Biosystems, Brazil) and 5 µl of the above described DNA eluate, in a final volume of 25 µL.

**Table 1:** Primers and probe used in the TaqMan assay for HHV-8 DNA.

	Sequence 5′ 3′	GenBank position^a^
Forward primer	GGGCCCCGGATGATGTA	137
Reverse Primer	GCCCCATAAATGACACATTGG	182
Probe	6- *FAM*-AGATCAAGTTCCGCCATAT –*MGB*	157

a According to Genbank entry: AF042371.1

Cycling conditions consisted of two initial heat-activation steps of 50°C for 2 minutes (UNG) and 95°C for 10 minutes (Taq polymerase), followed by 40 cycles of 15 seconds at 95°C and 1 minute at 65°C, in an ABI 7300 real-time PCR instrument. Probit analysis was performed using the proportion of experimental points displaying a positive result, i.e. Ct below the cut-off automatically established by the real-time PCR instrument. The concentration of 1,000 copies/mL was determined by this analysis to present a hit rate of 95%, corresponding to a cycle threshold (Ct) of 36 (Table 2).

**Table 2:** Results of replicate testing of whole blood spiked with known amounts of plasmid containing HHV-8-ORF 26.

Plasmid copies/mL	Number of positive replicates/Number of tested replicates
19500	10/10
1950	10/10
975	9/10
487.5	8/10
243.7	7/10
195	2/10
121.8	2/10
60.9	1/10
12	0/10
1.2	0/10

#### Specificity

The assay was challenged against DNA from CMV, HSV-1, HSV-2, EBV, VZV and HHV-6 derived from cell lines and standards with no amplification.

#### Run control

DNA isolated from the cell line BCBL-1, previously demonstrated to contain 36 copies of HHV-8 per cell [17] was used as a positive run control on each experiment. A blank (water) tube was included in each run as a negative control.

### ORF 73 real time PCR

Real-time PCR for this target was performed under the same conditions as above. Primers and probe were described by Krishnan *et al*. [18]. A plasmid pGEX-5X containing an insert from the ORF 73 (generous gift of Dr. Harutaka Katano, Laboratory of Pathology, AIDS Research Center, National Institute of Infectious Diseases, Tokyo, Japan) was used to determine the limit of sensitivity, which was of 700 copies/mL of whole blood at the 95% detection rate.

## Results

β-globin could be amplified in 803 (91.5%) of the 878 ELISA-positive samples, indicating adequate amounts of purified DNA and removal of PCR inhibitors. HHV-8 DNA was not detected in any of the valid 803 whole-blood samples after triplicate PCR testing. In 48/803 (6%) of the samples, discrepancies were observed among the replicates in the real-time PCR assay (one positive in three), always at Ct values above the established threshold of 36. Since this could represent donors harboring low viral loads (<1,000 copies/mL), we repeated the analysis and also submitted the DNA to amplification of another region (ORF73). A positive amplification was never reproducible in the same assay nor corroborated by the ORF73 real-time PCR assay for any of these samples and the 48 donors were considered as HHV-8 DNA negative.

## Discussion

Almost 20 years after the discovery of HHV-8 by Chang *et al*. [18] there is still no consensus about its potential transmission by blood transfusion. In this study, despite testing a large number of HHV-8 seropositive blood donors by a stringent protocol of repeat and complementary PCRs, HHV-8 DNA could not be consistently detected in any blood sample.

The decision to test blood donors shown to have HHV-8 antibodies after screening with a whole virus ELISA was stimulated by a previous report from Uganda showing a significantly higher risk of seroconversion among recipients of HHV-8-seropositive blood units compared to recipients of HHV-8 seronegative blood [11]. The choice of appropriate serological screening tests to detect HHV-8 infection, particularly in apparently healthy individuals, is problematic due to the lack of a gold-standard to ascertain the true status of infection. Routine HHV-8 screening of blood donors is not currently performed anywhere in the world. However, since HHV-8 transmission by blood transfusion is biologically plausible, screening of blood units might be considered in populations with intermediate to high HHV-8 seroprevalence, especially when destined to be used for immunocompromised recipients. In this case, recipients' safety would argue for the use of highly sensitive tests. The HHV-8 whole-virus ELISA used in this study, as well as other enzyme immunoassays based on peptide-containing antigenic HHV-8 gene products, such as the lytic HHV-8 glycoprotein K8.1, have been shown to have excellent sensitivity, making them suitable for HHV-8 screening of blood donors [19]; [20].

The lack of detection of HHV-8 DNA in any of the 803 whole blood samples from HHV-8 seropositive blood donors in the present study corroborates findings of previous studies carried out in low prevalence populations, but is in stark contrast with reports from intermediate to high prevalence regions. In Italy, Bigoni *et al*. [21] detected HHV-8 DNA in 5/56 (9%) PBMCs from blood donors, while Brown *et al*. [22] detected HHV-8-DNA in 16.5% of PBMCs from 158 HHV-8 seropositive healthy adults, and Vitale *et al*. detected HHV-8 DNA in the PBMCs of one out of 12 healthy HHV-8 seropositive individuals [23]. In Tanzania [3] and the Central African Republic [9], HHV-8 DNA was detected in 4.5% and 22.5% of blood donors, respectively. Conversely, our results accord with studies undertaken among healthy blood donors from low prevalence areas such as the USA. Hudnall *et al*. [4] evaluated 23 buffy-coat samples from HHV-8 seropositive blood donors using nested PCR with primers specific for the ORF26 minor capsid gene and the HHV-8 ORF72 v-cyclin gene, but did not detect HHV-8 DNA in any sample. The same group tested 100 whole blood samples using a real-time PCR assay with TaqMan probes and did not detect any HHV-8 DNA [13]. Pellet *et al*. [12], performing nested PCR on whole blood samples from 138 donors including 55 who were seropositive for HHV-8, also failed to identify HHV-8 DNA in any sample. More recently, Qu *et al.* [6], testing purified DNA equivalent to more than 3×10^4^ B-cells from 861 donors, including 40 samples from HHV-8 seropositive individuals, did not detect HHV-8 DNA in any of these samples. Furthermore, HHV-8 genomes were not detected from induced/cultured PBMCs and CD19+ B-cells from 164 blood donors, including seven HHV-8 seropositive samples [24]. Unfortunately, in our work, we could not isolate PBMCs from whole blood samples, as repository samples were immediately frozen after whole blood collection at the blood bank. This could have helped concentrate DNA and increased assay sensitivity. Moreover, the freezing process may have led to some specimen damage, since in 9% of our samples we failed to amplify β-globin. Such sample inadequacy rate is not uncommon when working with frozen samples. However, DNA concentration was measured in a subset of 300 randomly chosen samples and only three samples (1%) had no OD values, indicating that the complete lack of HHV-8 DNA detection could not be entirely attributed to these technical limitations.

The consistent lack of HHV-8 DNA detection in blood donors from countries with low HHV-8 seroprevalence might be explained by the low number of seropositive donors enrolled in such investigations. However, even when only seropositive healthy individuals have been considered, striking differences between non-endemic and endemic regions have persisted. One possible hypothesis would be that, in endemic regions where there is an active viral circulation, acute infections with lytic viral replication and higher viral DNA copy numbers in the blood may not be uncommon. Conversely, in non-endemic countries, healthy immunocompetent HHV-8-seropositive individuals may harbor predominantly HHV-8 latently-infected cells that would not undergo productive (lytic) replication, unless in response to specific triggers. However, even when both lytic replication and latency occur concomitantly, such as demonstrated in HHV-8 seropositive individuals co-infected with HIV, HHV-8 viral loads may be low and frequently below the threshold of detection [25]. A recent study that included 40 HHV-8 seropositive blood donors and which enhanced the performance of a very sensitive quantitative real-time PCR (detection limit 1-6 copies) by purifying C19+ B-lymphocytes corroborated the notion that, in US blood donors, the prevalence of HHV-8 genomes in the circulatory blood is extremely low and generally undetectable [6]. However, we must acknowledge that the PCR method described in this study has a lower sensitivity when compared to nested PCR methods adopted in other studies. The aforementioned studies using PBMCs from healthy Italian volunteers were able to detect HHV-8 DNA sequences in 9 to 16% of the samples using PCR methods with a threshold of approximately 3 copies of HHV-8 DNA per 10^6^ cells. Our positivity cut-off was 1,000 copies/mL. If one considers that leukocyte counts on healthy blood donors average 5 to 10×10^6^ cells/mL, our method would classify as ‘positive’ donors harboring between 100 and 200 copies of HHV-8 DNA per 10^6^ cells. Viral loads of PBMCs from a control Italian group (HIV-seronegative non-KS patients) were shown to range from 13 to 2,128 copies/million cells [26]. Consequently, our study cannot entirely rule out the possibility of donors harboring very low HHV-8 viremia. Nevertheless, our findings may alleviate concerns over the need to screen donors for HHV-8 in Brazil and do not support the introduction of routine HHV-8 screening in Brazilian blood banks, since, in the current context, HHV-8 represents a low-level infectious disease threat to our blood supply [27].
